# Radiomic- and dosiomic-based clustering development for radio-induced neurotoxicity in pediatric medulloblastoma

**DOI:** 10.1007/s00381-024-06416-6

**Published:** 2024-04-20

**Authors:** Stefano Piffer, Daniela Greto, Leonardo Ubaldi, Marzia Mortilla, Antonio Ciccarone, Isacco Desideri, Lorenzo Genitori, Lorenzo Livi, Livia Marrazzo, Stefania Pallotta, Alessandra Retico, Iacopo Sardi, Cinzia Talamonti

**Affiliations:** 1 Department of Experimental and Clinical Biomedical Sciences “Mario Serio”, University of Florence, Florence, Italy; 2 National Institute for Nuclear Physics (INFN), Florence Division, Florence, Italy; 3 Radiation Oncology Unit, Careggi University Hospital, Florence, Italy; 4 Radiology Unit, Meyer Children’s Hospital IRCCS, Florence, Italy; 5 Medical Physics Unit, Meyer Children’s Hospital IRCCS, Florence, Italy; 6 Neuro-Oncology Unit, Meyer Children’s Hospital IRCCS, Florence, Italy; 7 Pisa Division, INFN, Pisa, Italy

**Keywords:** Small data, Radiomics, Dosiomics, Pediatric medulloblastoma, Neurotoxicity

## Abstract

**Background:**

Texture analysis extracts many quantitative image features, offering a valuable, cost-effective, and non-invasive approach for individual medicine. Furthermore, multimodal machine learning could have a large impact for precision medicine, as texture biomarkers can underlie tissue microstructure. This study aims to investigate imaging-based biomarkers of radio-induced neurotoxicity in pediatric patients with metastatic medulloblastoma, using radiomic and dosiomic analysis.

**Methods:**

This single-center study retrospectively enrolled children diagnosed with metastatic medulloblastoma (MB) and treated with hyperfractionated craniospinal irradiation (CSI). Histological confirmation of medulloblastoma and baseline follow-up magnetic resonance imaging (MRI) were mandatory. Treatment involved helical tomotherapy (HT) delivering a dose of 39 Gray (Gy) to brain and spinal axis and a posterior fossa boost up to 60 Gy. Clinical outcomes, such as local and distant brain control and neurotoxicity, were recorded. Radiomic and dosiomic features were extracted from tumor regions on T1, T2, FLAIR (fluid-attenuated inversion recovery) MRI-maps, and radiotherapy dose distribution. Different machine learning feature selection and reduction approaches were performed for supervised and unsupervised clustering.

**Results:**

Forty-eight metastatic medulloblastoma patients (29 males and 19 females) with a mean age of 12 ± 6 years were enrolled. For each patient, 332 features were extracted. Greater level of abstraction of input data by combining selection of most performing features and dimensionality reduction returns the best performance. The resulting one-component radiomic signature yielded an accuracy of 0.73 with sensitivity, specificity, and precision of 0.83, 0.64, and 0.68, respectively.

**Conclusions:**

Machine learning radiomic-dosiomic approach effectively stratified pediatric medulloblastoma patients who experienced radio-induced neurotoxicity. Strategy needs further validation in external dataset for its potential clinical use in ab initio management paradigms of medulloblastoma.

**Supplementary Information:**

The online version contains supplementary material available at 10.1007/s00381-024-06416-6.

## Introduction

Medulloblastoma (MB) is the most common brain malignancy in pediatric patients, which accounts for 20–25% of pediatric central nervous system neoplasms [1, 2]. Despite the increase in survival rates in recent years, prognosis of MB patients remains relatively poor, and it strongly depends on clinical and molecular risk factors [3].

In the past, the risk stratification was based on age at diagnosis, disease dissemination, and extent of resection. Recently, a new proposed classification identifies four risk categories (low, standard, high, and very high risk) taking into account metastatic stage and genetic and cytogenetic aberrations characterized by very different clinical outcomes and treatment resistance [4]. MB is currently treated with surgery, chemotherapy, and craniospinal irradiation (CSI). Cure intensification is based on risk stratification and despite this multimodal approach, about 30% of high-risk patients experience disease relapse [5].

Moreover, due to the aggressive therapies and the young age of MB patients, early and late sequelae such as ototoxicity, cardiotoxicity, lung toxicity, neurotoxicity, endocrine deficiency, and neurocognitive deficits could often develop [6–8]. In particular, neurotoxicity could compromise quality of life in pediatric patients; for example, long-term neurological sequelae imply that children treated with high dose chemotherapy and/or radiotherapy for central nervous system tumors had lower educational outcomes [9]. The factors that concur to develop neurotoxicity in pediatric patients are argument of scientific discussion; in a recent retrospective review of 113 patients treated with CSI for medulloblastoma, the authors showed a dose response relationship between radiotherapy and neurocognitive impairment [10]. New radiotherapy technique, smaller radiotherapy field, and lower dose are investigated to reduce the impact of radiotherapy on neurotoxicity in central nervous system tumors in children [11]. Furthermore, the improvement of diagnostic imaging led to magnetic resonance imaging (MRI) becoming the gold standard in central nervous system tumors [12]. Due to the high resolution of morphologic images, MRI guides the clinician with the differential diagnosis and consequently the first approach to the therapeutic path; moreover, multiparametric MRI is useful to define treatment response not only detecting tumor shrinkage but also to distinguish pseudo progression and early signs of neurotoxicity [13].

A revolutionary approach to medical imaging has been done with radiomics. The imaging analysis allows the extraction of quantitative features that could be used for clinical purposes. Radiomics derived data when used in combination with clinical data could offer information not only about cancer genotype but also clinical outcome and toxicity treatment correlated [14–17]. We hypothesize that non-invasive biomarkers offer great potential for improving stratification in pediatric medulloblastoma. The aim of this study is to analyze MRI features of metastatic MB patients treated with surgery, chemotherapy, and CSI and look for quantitative features that correlate with clinical outcome. Moreover, the correlation between MRI radiomics features and dosimetric distribution on planning computed tomography (CT) are investigated to predict radio-induced neurotoxicity. This toxicity has been identified with radio-necrosis, a condition characterized by the death of tissue due to exposure to high doses of radiation and frequently occurs in the brain. The combination of radiomics and dosiomics (i.e., to extract texture features from dose distribution [18]) analysis is likely to provide non-invasive imaging biomarker of clinical outcome and radio-induced toxicity.

## Methods

### Dataset

All procedures performed in this study involving human participants were in accordance with the ethical standards of the institution and the Declaration of Helsinki (as revised in 2013). The study was approved by the Institutional Pediatric Ethics Committee. Since this study is a retrospective analysis and the patients have been anonymously processed, the need for informed consent was waived.

In this single-center analysis, data of patients referred for adjuvant radiotherapy for pathologically confirmed primary MB patients were initially analyzed for further inclusion. The inclusion criteria were (i) availability of postoperative MRI with diagnostic-quality performed after adjuvant radiotherapy throughout the follow-up period, (ii) availability of multi-parametric MRI, including axial T1-weighted, T2-weighted, and FLAIR maps, (iii) availability of radiotherapy CT, structures set, plan, and 3D dose volume. Patients with incomplete clinical data, poor tumor tissue quality, and incomplete or poor-quality MR images were excluded from the research. Baseline demographic clinical information including age, gender, metastasis, histologic subtype, and adjuvant therapies (radiation alone, chemotherapy alone or both of them) were collected from the medical record system.

Regarding the follow-up data, obtained by medical records, the clinical practice requires MRIs to be acquired 30–45 days within the last day of radiotherapy treatment and every 3 months for the first 3 years after surgery and every 6 months thereafter. During the follow-up, neurological and endocrine assessment were recorded. This has allowed clinicians to identify whether or not the radio-induced neurotoxicity has occurred and to obtain the ground truth label, hereinafter referred to as “relapse.” While for radiotherapy-treatment, patients treated with craniospinal irradiation received to the entire brain and spine either standard-dose (i.e., 39 Gy) or reduced-dose (i.e., 31.2 Gy) in patients < 10 years old. In some cases, patients received a boost to the entire posterior fossa or a focal conformal boost to tumor bed if a residual disease is present; in both cases, total boost volume dose was up to 60 and 59.7 Gy in patients > 10 or < 10 years old, respectively. Moreover, the radiation was delivered with helical Tomotherapy [19].

### Radiomic feature extraction

**Fig. 1:**
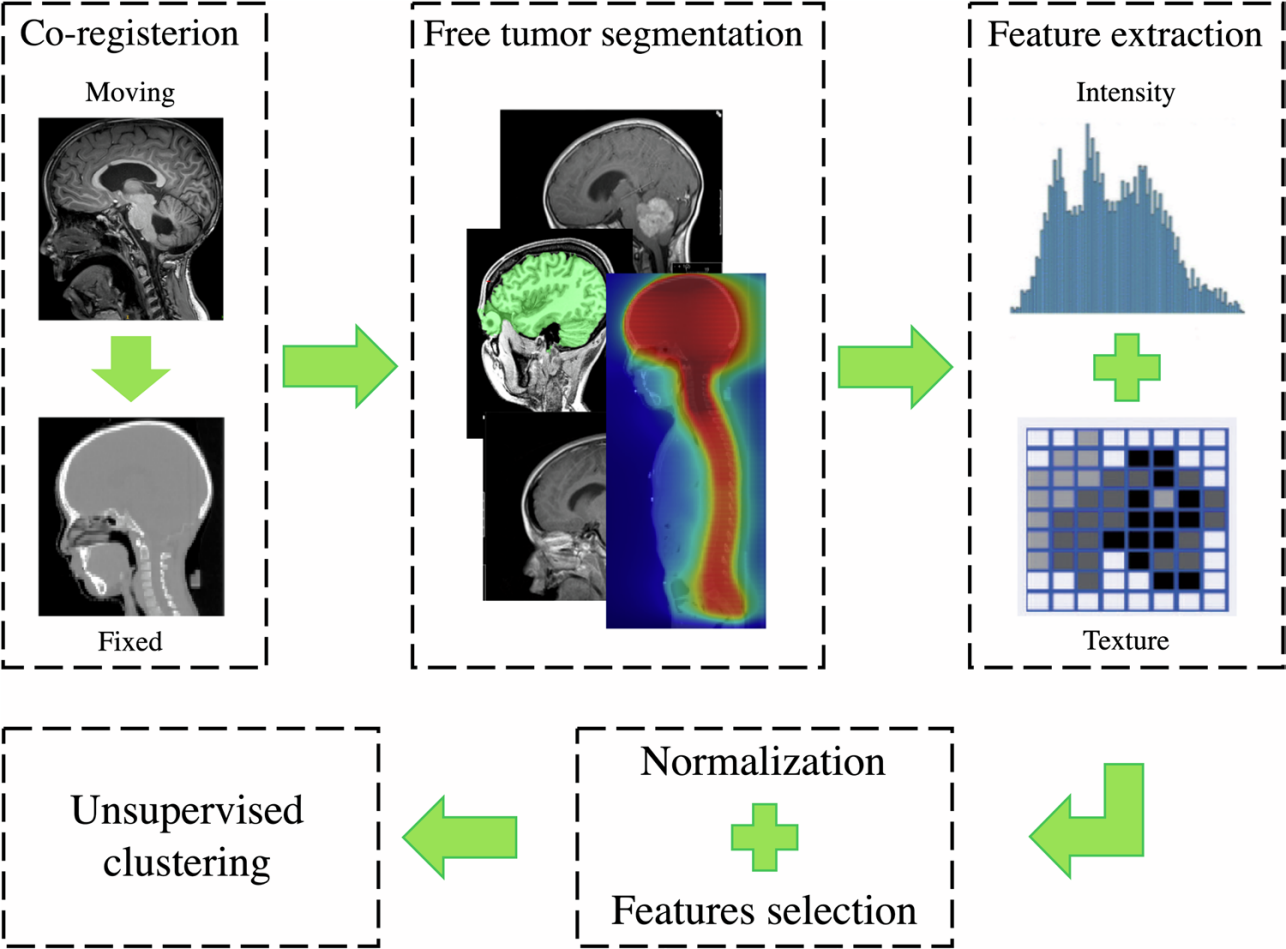
Radiomics workflow pipeline

Prior to feature extraction, two fundamental data pre-processing steps were carried out across all the patients: resolution adjustment and images co-registration. Three-dimensional tumor contours were obtained free from radiotherapy process by co-registration of MR images on the centering CTs. Pixels included by the defined tumor contour were applied for feature extraction using PyRadiomics (v2.2.0) [20], an open-source Python tool. A detailed description of the implementation of these steps and radiomic features extracted by the software is available in the official documentation (https://pyradiomics.readthedocs.io/en/latest/features.html). A diagram illustrating images processing and the overall workflow is displayed in Fig. 1.

Features quantifying tumor phenotypic characteristics on MR and dose images could be grouped as tumor intensity and texture features. In the first category, tumor intensity information is quantified using first-order statistics, obtained from the histogram of entire tumor voxel intensity values. While the second category consists of three-dimensional texture features that are able to quantify the intra-tumoral heterogeneity within a full tumor volume. Textural features were computed based on Gray Level Cooccurrence Matrix (GLCM), Gray Level Run Length Matrix (GLRLM), Gray Level Size Zone Matrix (GLSZM), Gray Level Dependence Matrix (GLDM), and Neighboring Gray Tone Difference Matrix (NGTDM).

### Feature selection and classifiers

Data mining and machine learning analysis were performed in the Colab environment (https://colab.research.google.com).

To reduce the batch effects, in the feature analysis, the quantitative radiomics raw data were normalized across all patients. Two different feature selection and ranking method were employed in the analysis based on multivariate filter approaches and on recursive feature elimination. Filter methods are feature-ranking methods, which rank the features using a scoring criterion, and multivariate methods investigate the multivariate interaction within the features and the scoring criterion is a weighted sum of feature relevancy and redundancy. Feature relevancy is a measure of feature’s association with the target/outcome variable, whereas feature redundancy is the amount of redundancy present in a particular feature with respect to the set of already selected features [21]. The second approach concerns recursive feature elimination (RFE). It aims to identify the most relevant features from a given dataset by iteratively eliminating less important features based on their contribution to a model’s performance. Given an external estimator that assigns weights to features, the algorithm recursively eliminates least important features considering smaller and smaller sets of features. The number of features to eliminate at each iteration is a parameter that needs to be specified. After removing the least important features, the model is retrained on the reduced feature set. That procedure is recursively repeated on the pruned set until a predetermined number of features remain or until a specific stopping criterion is met. We exploited mutual information (MI) and RFE in a fivefold cross-validation fashion for feature selection and ranking, and principal component analysis (PCA) as dimensionality reduction.

We implemented three different classification methods: Random Forest (RF), Extreme Gradient Boosting (XGB), and Hierarchical Clustering (HC), and external cluster validation method was applied to get the prediction accuracy. We want to spend a few words about the last two less common classifiers and deepen these concepts of data mining.

Extreme Gradient Boosting [22] is designed to solve supervised learning problems, and it is an enhanced version of the traditional gradient boosting algorithm. An ensemble model combines the outputs of multiple weak prediction models to create a stronger and more accurate model. The Random Forest is a popular ensemble that takes the average of many decision trees via bagging. Bagging is short for “bootstrap aggregation,” meaning that samples are chosen with replacement (bootstrapping) and combined (aggregated) by taking their average. Boosting is a strong alternative to bagging. Instead of aggregating predictions, boosters turn weak learners into strong learners by focusing on where the individual models went wrong. In Gradient Boosting, individual models train upon the residuals which are the difference between the prediction and the actual results. Instead of aggregating trees, gradient-boosted trees learn from errors during each boosting round. The key idea behind XGB is to optimize a specific loss function by iteratively adding weak models and updating the model’s predictions based on the residuals. The “eXtreme” refers to speed enhancements since it supports parallel computing. In addition, XGB includes a unique split-finding algorithm to optimize trees, along with built-in regularization to prevent overfitting and improve generalization and which controls the complexity of the model.

Hierarchical cluster analysis is an unsupervised clustering algorithm. The algorithm groups similar objects into groups called clusters. The endpoint is a set of clusters or groups, where each cluster is distinct from each other cluster, and the objects within each cluster are broadly similar to each other. Clustering technique is based on measures of similarity between pair of items in the data set. This similarity is conceived in terms of distance in a multidimensional space, such as the Euclidean distance. Clustering algorithm then group the elements on the basis of their mutual distance, specifically it works out which observations to group based on reducing the sum of squared distances of each observation from the average observation in a cluster. Therefore, whether or not the elements belong to a set depends on how far the element under consideration from the set is. The main advantage of hierarchical clustering is that the number of clusters does not have to be defined a priori. Moreover, this technique can be displayed in an attractive, tree-based representation of the observations, called a dendrogram. The tree is not a single set of clusters, but rather a multilevel hierarchy, where clusters at one level are joined as clusters at the next level. The distance between data points represents dissimilarities, while height of the blocks represents the distance between clusters.

Concerning cluster validation, external clustering validity approach uses prior knowledge and consists in comparing the results of a cluster analysis to an externally known result, such as externally provided class labels. It measures the extent to which cluster labels match pre-existing clustering structure (reference labels). We preferred this method since we know the “true” cluster number and reference labels in advance [23]. Measures of the machine learning classifier performance included: accuracy, sensitivity (recall), specificity, precision, F1-score, and Matthews Correlation Coefficient (MCC).^1^

## Results

From September 2011 to November 2019, data of 48 medulloblastoma patients treated with CSI were collected. The mean age was 12 ± 6 years (range, 2–23 years); 29 (60.4%) patients were males. At a mean follow-up of 54 months (2–96 months), 37 (77%) patients were alive. Twenty-nine (60.4%) patients had a disease recurrence after CSI; the mean time to recurrence from the date of the last radiotherapy treatment was 21 months (range, 1–49). During the follow-up, 26 (54%) patients developed a pituitary hypopituitarism; in particular, 11 (23%) had low cortisol, 7 (14.6%) hypothyroidism, and 8 (16.7%) pan hypopituitarism. The mean time of pituitary hypopituitarism onset was 21 months (range, 12–48). During the follow-up, at the neurocognitive evaluation, in 11 (23%) patients, neurocognitive deficits were recorded at a mean time of 23 months. Indeed, 3 (6%) patients had working memory deficit and 8 (16.7%) patients developed attention deficit. At the periodic clinical evaluation in 7 (14.6%) patients, neuromotor deficits were recorded, 3 patients developed tetraplegia, 3 ataxias, and 1 hemiparesis; the neurological deficit was diagnosed at the time of radiological progression in 3 patients. At a mean time of 16 months (range, 3–48), 40 (83%) patients developed radiological evidence of neurotoxicity. At last follow-up, two patients had radiological evidence of radionecrosis and one patient had cerebral edema. All the patients with neurological toxicity had radiological neurodamage. Starting from diagnostic radiology, extractions of a total of 332 radiomic and dosiomic features have been performed from each patient, 83 for each of the four available images series (three MRI sequences and dose distribution). Their pairwise correlation cluster map can be found in supplementary file 1. Among these, feature selection worked by selecting the k best most informative features based on MI statistical test. In our case, the 20 best features derived from dose, T1w and T2w images, and FLAIR maps are made explicit in Table 1. Their univariate and bivariate distribution in our population based on relapse occurrence can be found in supplementary file 2.

**Table 1:** Characteristics of each selected feature and relative class according to PyRadiomics official documentation (https://pyradiomics.readthedocs.io/en/latest/features.html)

**Sequence**	**Features**	**Features class**	**Acronym**
Dose	Small area high gray level emphasis	Gray Level Size Zone Matrix	SAHGLE_glszm_Dose
	Zone entropy		ZE_glszm_Dose
	Inverse difference moment normalized	Gray Level Co-occurrence Matrix	IDMN_glcm_Dose
	Busyness	Neighboring Gray Tone Difference Matrix	Busyness_ngtdm_Dose
	Small dependence high gray level emphasis	Gray Level Dependence Matrix	SDHGLE_gldm_Dose
T1w	Maximal correlation coefficient	Gray Level Co-occurrence Matrix	MCC_glcm_T1
	Sum square		SS_glcm_T1
	Gray level variance	Gray Level Dependence Matrix	GLV_gldm_T1
	Gray level variance	Gray Level Run Length Matrix	GLV_glrlm_T1
	Coarseness	Neighboring Gray Tone Difference Matrix	Coarseness_ngtdm_T1
T2w	Gray level variance	Gray Level Dependence Matrix	GLV_gldm_T2
	Difference average	Gray Level Co-occurrence Matrix	DV_glcm_T2
	Difference entropy		DE_glcm_T2
	Difference variance		DA_glcm_T2
	Contrast		Contrast_glcm_T2
	Cluster tendency		CT_glcm_T2
	Sum squares		SS_glcm_T2
	Gray level non-uniformity normalized	Gray Level Run Length Matrix	GLNUN_glrlm_T2
	Gray level variance		GLV_glrlm_T2
FLAIR	Busyness	Neighboring Gray Tone Difference Matrix	Busyness _ngtdm_FLAIR

**Fig. 2:**
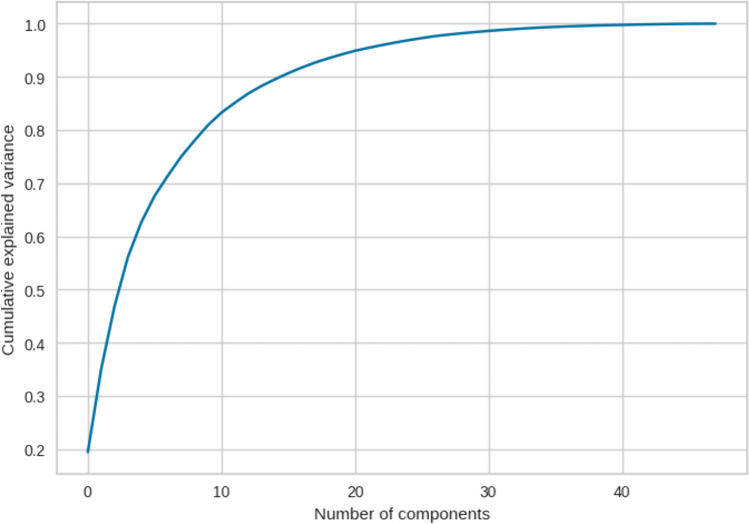
Cumulative explained variance as function of the number of features. Already with 20 features, it is possible to be accounted for 95% of the total variance

**Fig. 3:**
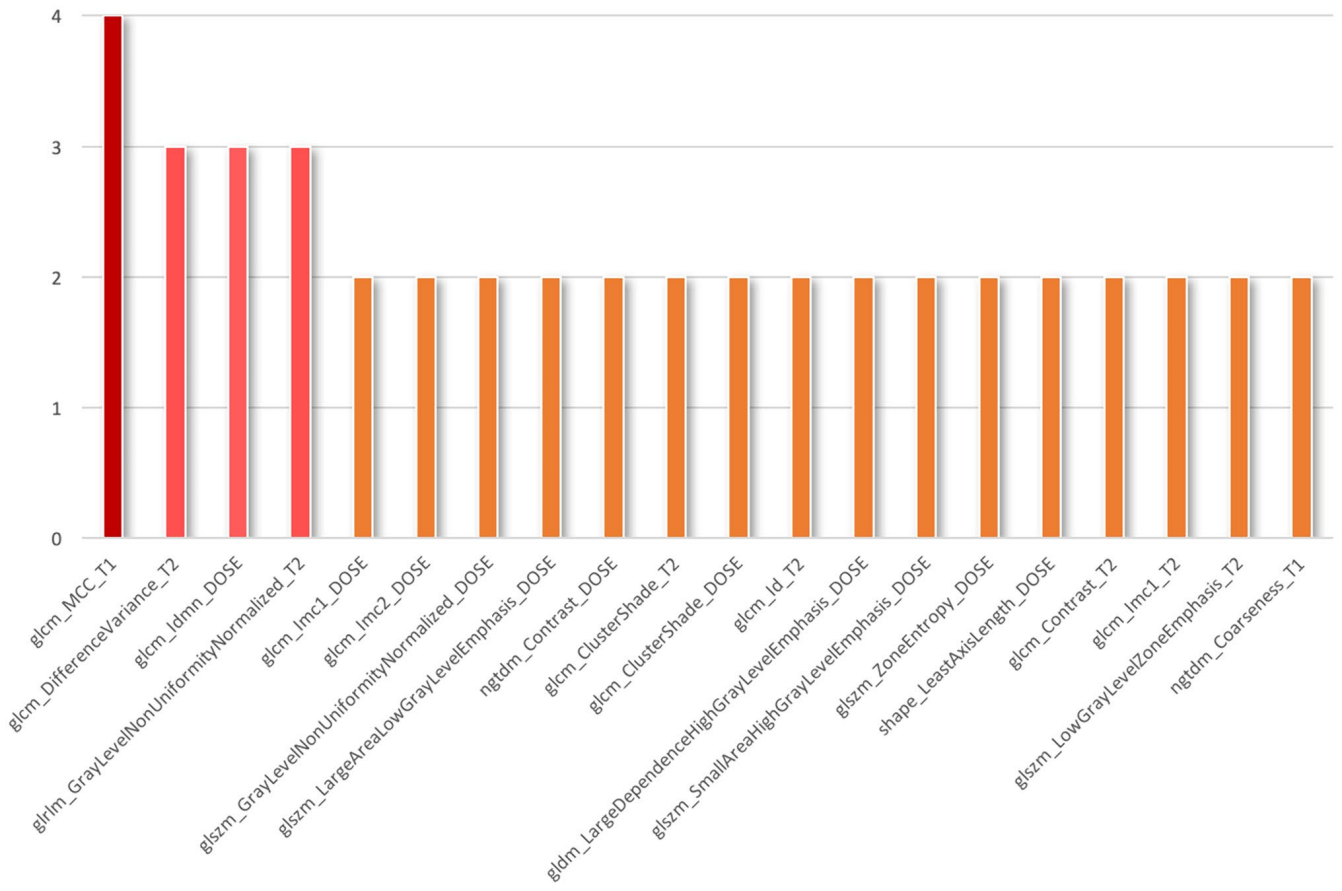
Histogram of the frequency of the top features identified with the different selection methods

The first step of our strategy for feature selection was to consider the best 20 features (correlation matrix can be find in supplementary file 3) based on the explained variance; indeed, in Fig. 2, it can be seen how already with only 20 components (intended as the number of features), it is possible to maintain as much as 95% of the variability present in the data. A higher explained variance indicates a better fit and suggests that the model is capturing a significant portion of the underlying relationships between the variables. Taking into account the second selection and ranking method, in RFE, we set the achievement of 20 features as a stopping criterion following what we learned a little while ago. The process was repeated with four common external estimators (Logistic Regression, LR; Decision Tree, DT; Random Forest, RF; Gradient Boosting, GB), and the selected features were compared with those identified by MI statistical test. From the histogram in Fig. 3, it is possible to notice how certain variables are more frequently present in the subsets of 20 features and are also the same ones that are found in the first places of the ranking proposed by the MI analysis.

As second step, feature reduction was conducted exploiting the PCA technique, which permits a dimensionality reduction. It combines input data by projecting them into a lower number of components, four and one in our case, following the rule of thumb to select 1 feature every 10/15 variables. Thereby, we increase the informative power of the remaining features, but we cannot have a direct definition of what each single feature describes.

The evaluation metrics according to the various strategies for features selection and features reduction are shown in the Table 2. Considering the best performing strategy, its accuracy is 0.73 with 35/48 correctly classified patients. In particular, analyzing sensitivity and specificity, the model demonstrates good prediction power at identifying patients who have suffered radio-induced toxicity.

**Table 2:** Classifier evaluation metrics. MCC, Matthews correlation coefficient

	**Explained variance**	**Model**	**Accuracy**	**Sensitivity**	**Specificity**	**Precision**	***F*-score**	**MCC**
**All**	1.00	HC	0.56	0.16	1.00	1.00	0.28	0.29
		RF	0.58	0.64	0.52	0.59	0.62	0.16
		XGB	0.48	0.58	0.38	0.48	0.53	-0.04
**20-best**	0.95	HC	0.60	0.56	0.65	0.64	0.60	0.21
		RF	0.65	0.80	0.48	0.63	0.70	0.29
		XGB	0.63	0.68	0.57	0.63	0.65	0.25
**4-best**	0.67	HC	0.65	0.36	0.96	0.90	0.51	0.39
		RF	0.69	0.88	0.48	0.65	0.75	0.39
		XGB	0.65	0.68	0.61	0.65	0.67	0.29
**20-best → 4-PCA**	0.91	HC	0.63	0.54	0.71	0.65	0.59	0.25
		RF	0.67	0.81	0.50	0.66	0.72	0.32
		XGB	0.67	0.72	0.61	0.67	0.69	0.33
**20-best → 1-PCA**	0.74	HC	0.63	0.64	0.62	0.58	0.61	0.25
		RF	0.65	0.80	0.48	0.63	0.70	0.29
		XGB	0.65	0.60	0.70	0.68	0.64	0.30
**4-best → 1-PCA**	0.67	HC	0.71	0.60	0.83	0.79	0.68	0.44
		***RF***	***0.73***	***0.83***	***0.64***	***0.68***	***0.75***	***0.47***
		XGB	0.67	0.61	0.72	0.67	0.64	0.33
**20-RFE—LR**	0.93	HC	0.63	0.40	0.87	0.77	0.53	0.30
		RF	0.60	0.68	0.52	0.61	0.64	0.20
		XGB	0.60	0.64	0.57	0.62	0.63	0.21
**20-RFE—DT**	0.89	HC	0.58	0.24	0.96	0.86	0.38	0.28
		RF	0.60	0.76	0.43	0.59	0.67	0.21
		XGB	0.58	0.68	0.48	0.59	0.63	0.16
**20-RFE—RF**	0.91	HC	0.42	0.32	0.52	0.42	0.36	-0.16
		RF	0.69	0.88	0.48	0.65	0.75	0.39
		XGB	0.71	0.76	0.65	0.70	0.73	0.42
**20-RFE—GB**	0.92	HC	0.54	0.60	0.48	0.56	0.58	0.08
		RF	0.67	0.80	0.52	0.65	0.71	0.34
		XGB	0.69	0.64	0.74	0.73	0.68	0.38

## Discussion

In recent years, an increasing number of reports demonstrated the added value of machine learning–based radiomics analysis to clinical and conventional MRI characteristics in pediatric MB, pointing out the potential to predict molecular markers and molecular subtype, to improve survival prediction, to evaluate the intratumoral heterogeneity, and to boost prognostic models [24–27]. However, to our knowledge, the relationships between the combination of radiomic-dosiomic features and radio-induced neurotoxicity of MB patients has not been investigated.

The main finding of this study is that our machine learning approach showed satisfactory stratification performance for clustering of pediatric medulloblastoma patients who have experienced radio-induced neurotoxicity based on radiomic and dosiomic features extracted from MR and dose images.

The accurate stratification of pediatric medulloblastoma patients is highly desired to select the most appropriate treatment [24], especially in view of a dose de-escalation with the same disease control. Indeed, patients treated with higher doses are prone to experience radio-induced neurotoxicity resulting in a worse intellectual outcome [10].

The machine learning protocol followed in this study foresees examining the dose distribution calculated for the radiotherapy treatment plans and the MR images of the first follow-up after radiotherapy. In this study, clinical outcome and neurological sequelae were reported, but the correlation between neurological deficits and radiomic and dosiomic features will be investigated in subsequent analyses. From the quantitative data extracted from these images, it was possible to establish a radiomic signature that has the potential to early highlight patients in whom radio-induced damage will develop. This could have a great clinical impact, because it gives the physician the possibility to intervene promptly with adequate therapies and reduce complications, since the detriments caused by ionizing radiations have a medium-to-long latency.

Considering the feature extraction and reduction strategies, it was possible to appreciate that a greater level of abstraction of input data by combining the selection of the most performing features and the reduction of dimensionality with PCA returns a better prediction performance. The best result was obtained by taking into consideration the 4-best features according to the ranking given by the MI test and projecting these four variables into a single component. In this scenario, satisfactory results of the various metrics were obtained for all three classifiers; in particular, the podium was awarded by the RF algorithm with an accuracy of 0.73 and an MCC of 0.47. This outcome is remarkable given the small number of the database and indicates a good agreement between the predicted and actual classifications, probably also due to the simplicity of the trained model which made it possible to contain overfitting. The resulting drawback of this approach is that we no longer have a direct definition of what each single feature describes.

Taking a step back and examining the description of the four best identified features, it can be seen how they consider small size zones with high gray-level values indicating dose hot-spots, disparity in intensity among neighboring voxels and heterogeneity across intensity levels for T2-weighted maps, and texture complexity in T1-weighted maps. All of them can be seen as describing two fundamental properties: homogeneity and heterogeneity of the underlying tissue and dose microstructure [28, 29]. Further, it must be highlighted that features extracted from dose images also contribute to the construction of the radiomic signature. In addition, following the scores presented in Fig. 3, it is confirmed that certain features are robust with respect to the various feature selection methods.

Taking into account the classification methods, all three techniques showed good results, comparable to each other and without running into overfitting. Between the two supervised algorithms, RF shows on average slightly better performance than XGB, probably due to for its simplicity, scalability, and robustness to noise, and therefore, it is possible to train better even with small data size available. From the results in Table 2, we can say that unsupervised clustering has intermediate performance respect to the two systems just described. It is necessary to point out that hierarchical clustering does not require any prior assumptions, and the classification we found arose spontaneously from the data without forcing. The hierarchical cluster tree may naturally divide the data into distinct, well-separated clusters. This can be particularly evident in the attractive dendrogram representation created from data where groups of objects are densely packed in certain areas and not in others (supplementary file 4).

To sum up, we obtained comparable performance applying two intrinsically different methods; on one hand, supervised learning algorithms learn patterns and relationships between features and target variable, on the other unsupervised learning algorithm groups similar data points into clusters based on their distances or similarities discovering inherent patterns without any predefined target variable. This indicates the goodness of the available data and the care taken in creating the database, albeit of modest dimensions.

Moreover, it is necessary to point out that satisfactory results were obtained despite the fact that the available database was small. Machine learning can be applied to small databases, although there are some considerations and challenges to keep in mind when working with limited data. Small databases may have a limited number of samples respect to the parameters to be optimized, which can lead to overfitting and make it challenging to build complex models. However, there are several techniques and best practices that can help address these issues and still achieve meaningful results. While applying machine learning to small datasets can be challenging, it is still possible to obtain valuable insights and predictions. The success of the chosen approach will depend on the careful selection of models, features, and techniques that are appropriate for the specific dataset and problem [30, 31].

Nonetheless, some limitations of this study need to be addressed. First, pediatric MB is a rare tumor, and although our research extends over 8 years, the patients’ cohort is quite limited. In addition, the data were all from a single institution although this peculiarity has allowed us to build a homogeneous, complete, and balanced database. Second, an external patient population for assessing the radiomics signature generalizability is not available. Future investigations will require data exchange between different institutions to obtain a higher volume database thanks to which it could be possible to obtain performance more reflective of the real predictive power of the current method.

## Conclusions

We believe the current imaging techniques may potentially be further equipped to better classify and safely diagnose possible complications and the current study demonstrated proof-of-concept results for integrating radiomics protocol. In this regard, radiomics and dosiomics may prove a valuable and cost-effective aid by providing non-invasive quantitative data that integrate qualitative image information already available.

### Supplementary Information

Below is the link to the electronic supplementary material.

Supplementary file1 (DOCX 2356 KB)

## Data Availability

The data that support the findings of this study are not openly available due to reasons of sensitivity and are available from the corresponding author upon reasonable request.

## Footnotes

1 MCC is a measure of the quality of binary classifications in machine learning, ranging from + 1 (perfect prediction) to 0 (average random prediction) and − 1 (inverse prediction).
