# Supplementary material for: Radiomic- and dosiomic-based clustering development for radio-induced neurotoxicity in pediatric medulloblastoma
Source: Childs Nerv Syst. 2024 Apr 20;40(8):2301–10. doi: 10.1007/s00381-024-06416-6 (PMC11269375; doi:10.1007/s00381-024-06416-6)
Supplement: Supplementary file 1 — Supplementary file1 (DOCX 2356 KB) [file 381_2024_6416_MOESM1_ESM.docx]

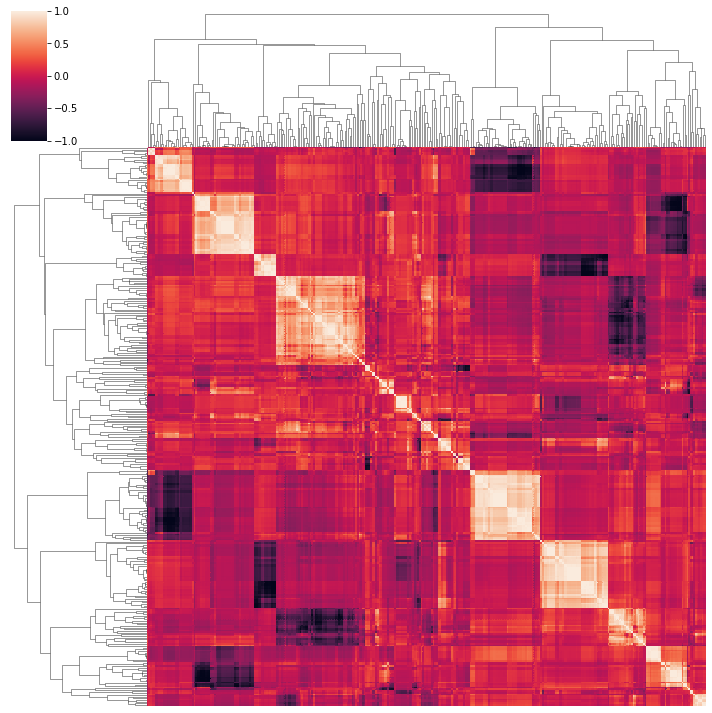


**Supplementary figure S1**. Pairwise correlation cluster map concerning all extracted features.


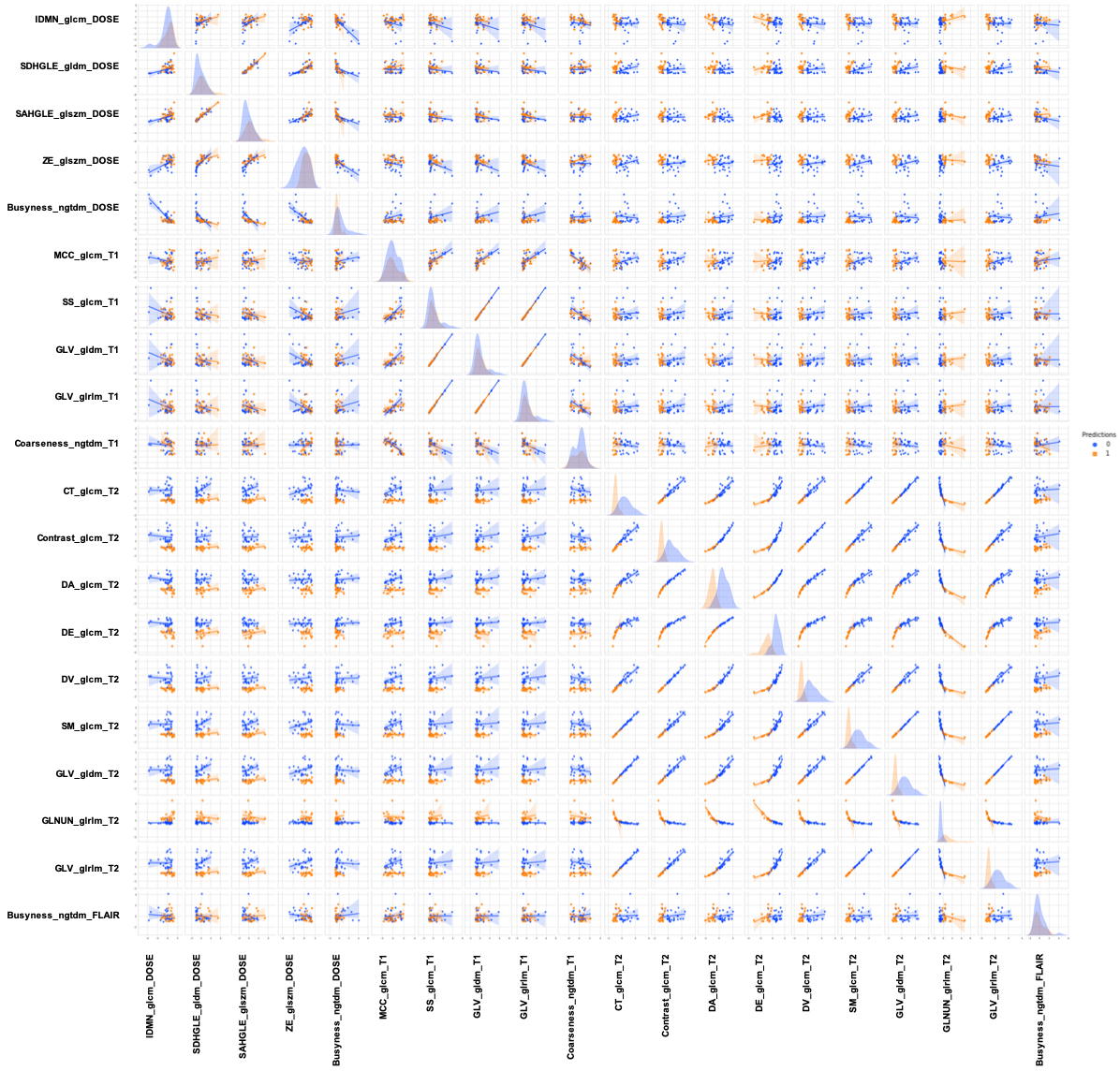


**Supplementary figure S2**. Univariate and bivariate distribution with regression lines for the 20-best selected features in relation to the relapse occurrence.


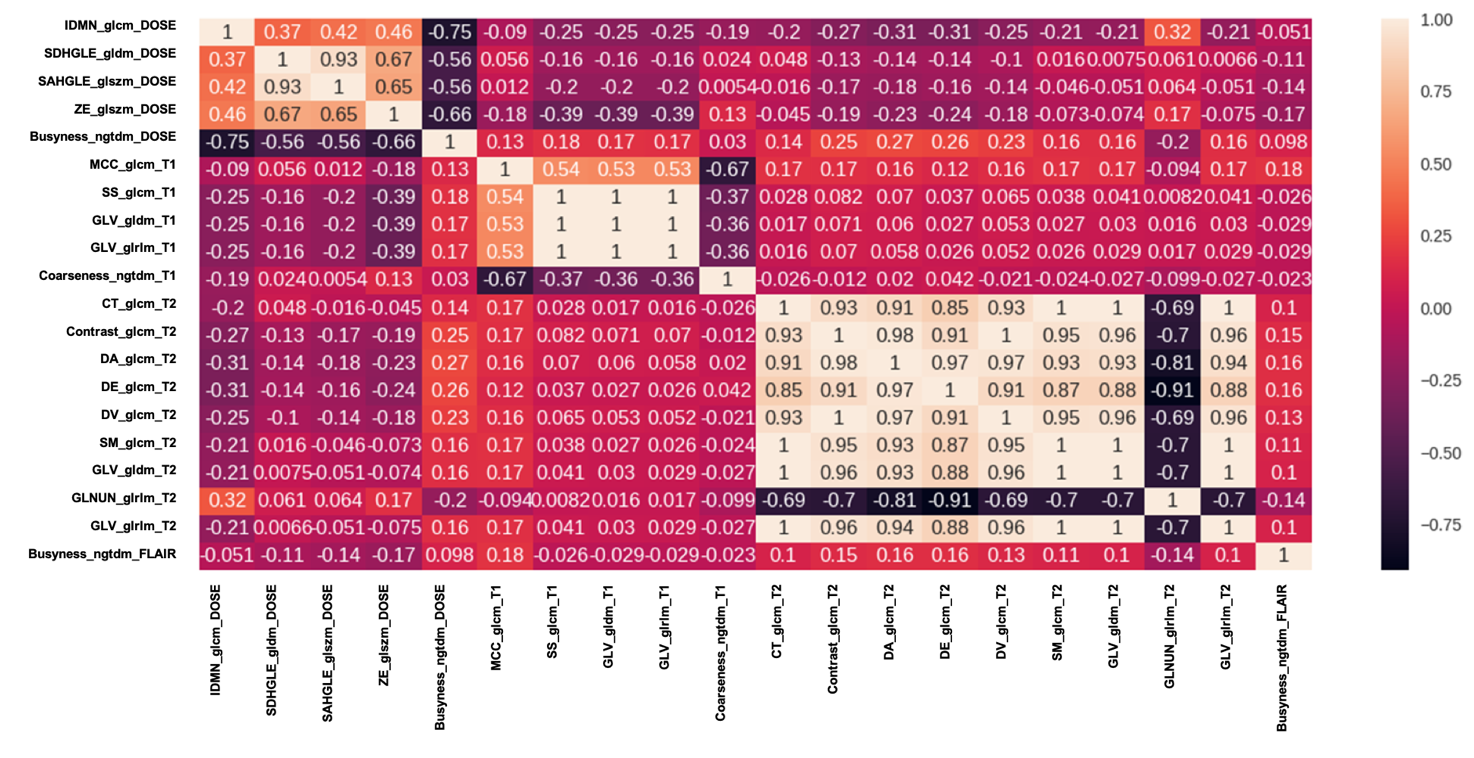


**Supplementary figure S3**. Correlation matrix of the 20-best extracted features.

**Supplementary figure S4**. Heat map of the reduced 4-best radiomics features signature. Hierarchical clustering with dendrogram of relapse occurrence is on the top. The red/blue bar indicates the true labels (red: yes, blue: no).
